# Supplementary material for: Hybrid Models and Biological Model Reduction with PyDSTool
Source: PLoS Comput Biol. 2012 Aug 9;8(8):e1002628. doi: 10.1371/journal.pcbi.1002628 (PMC3415397; doi:10.1371/journal.pcbi.1002628)
Supplement: Text S4 — Complete source code for the PyDSTool package (version 0.88.120504). Includes API documentation and help files linking to web pages. This file is identical to the current public release on Sourceforge.net. (ZIP) [file pcbi.1002628.s004.zip › PyDSTool/html/PyDSTool.Toolbox.PRCtools-module.html]

xml version="1.0" encoding="ascii"?


PyDSTool.Toolbox.PRCtools


| Home | Trees | Indices | Help | PyDSTool |
| --- | --- | --- | --- | --- |

Package PyDSTool ::
Package Toolbox ::
Module PRCtools

[hide private]

[frames] | no frames]

# Module PRCtools

source code

Toolbox for phase response curves measured by finite perturbations


|  |  |
| --- | --- |
| Functions | [hide private] |

|  |  |
| --- | --- |
| one\_period\_traj(model, ev\_name, ev\_t\_tol, ev\_norm\_tol, T\_est, verbose=False, initial\_settle=6, restore\_old\_ics=False, use\_quadratic\_interp=False)  Utility to extract a single period of a limit cycle of the model using forward integration, up to a tolerance given in terms of both the period and the norm of the vector of variables in the limit cycle at the period endpoints. | source code |

|  |  |
| --- | --- |
| \_default\_pert(model, ic, pertcoord, pertsize) | source code |

|  |  |
| --- | --- |
| finitePRC(model, ref\_traj\_period, evname, pertcoord, pertsize=0.05, settle=5, verbose=False, skip=1, do\_pert=<function \_default\_pert at 0x92efeb0>, keep\_trajs=False, stop\_at\_t=inf, force\_T=nan)  Pass a Generator or Model instance for model. | source code |

|  |  |
| --- | --- |
| compare\_pert(model, ref\_traj\_period, evname, pertcoord, pertsize, t0, settle=5, do\_pert=<function \_default\_pert at 0x92efeb0>, fignum=None)  Show perturbed and un-perturbed trajectories starting at t0, with given perturbation function do\_pert. | source code |

|  |  |
| --- | --- |
| fix\_PRC(PRC, tol=0.01)  Experimental and hopefully unnecessary utility to 'fix' PRC data with phase jumps of 1/2 or a whole cycle due to computational problems. | source code |


|  |  |
| --- | --- |
| Function Details | [hide private] |

|  |  |
| --- | --- |
| one\_period\_traj(model, ev\_name, ev\_t\_tol, ev\_norm\_tol, T\_est, verbose=False, initial\_settle=6, restore\_old\_ics=False, use\_quadratic\_interp=False) | source code |

Utility to extract a single period of a limit cycle of the model using
forward integration, up to a tolerance given in terms of both the period
and the norm of the vector of variables in the limit cycle at the period
endpoints.

Requires a non-terminal event in the model that is detected exactly
once per period. Assumes model initial conditions are already in domain
of attraction for limit cycle.

T\_est is an initial estimate of period. use\_quadratic\_interp option
(default False) indicates whether to make the returned trajectory
interpolated more accurately using quadratic functions rather than linear
ones. This option takes a lot longer to complete!

The model argument can be an instance of a Generator class or Model
class.

Returned trajectory will have name 'one\_period'.

|  |  |
| --- | --- |
| finitePRC(model, ref\_traj\_period, evname, pertcoord, pertsize=0.05, settle=5, verbose=False, skip=1, do\_pert=<function \_default\_pert at 0x92efeb0>, keep\_trajs=False, stop\_at\_t=inf, force\_T=nan) | source code |

```
Pass a Generator or Model instance for model.
Pass a Trajectory or Pointset for the ref_traj_period argument.
Pass the event name in the model that indicates the periodicity.
Use skip > 1 to sub-sample the points computed along the trajectory at
 the skip rate.
Use a do_pert function to do any non-standard perturbation, e.g. if there
 are domain boundary conditions that need special treatment. This function
 takes four arguments (model, ic, pertcoord, pertsize) and returns the new
 point ic (not just ic[pertcoord]).
Use settle=0 to perform no forward integration before the time window in
 which the perturbation will be applied (e.g. for non-cycles).
Use stop_at_t to calculate a partial PRC, from perturbation time 0 to this
 value.
Use force_T to force the period to be whatever value you like.

Note: Depending on your model, there may be regions of the PRC that are
offset by a constant amount to the rest of the PRC. This is a "wart" that
needs improvement.
```

  


| Home | Trees | Indices | Help | PyDSTool |
| --- | --- | --- | --- | --- |

|  |  |
| --- | --- |
| Generated by Epydoc 3.0.1 on Fri May 4 15:24:04 2012 | http://epydoc.sourceforge.net |
